# Physiologically-Based Pharmacokinetics of Ribociclib Drug–Drug Interactions and Organ Impairment Pharmacokinetics in Early Breast Cancer

**DOI:** 10.3390/ph19030461

**Published:** 2026-03-11

**Authors:** Yan Ji, Felix Huth, Craig Wang, Hilmar Schiller, Francois Pierre Combes, John Crown, Peter A. Fasching, Juan Pablo Zarate, Michael Untch

**Affiliations:** 1 Novartis Pharmaceuticals Corporation, East Hanover, NJ 07936, USA; 2 Novartis Pharma AG, CH 4056 Basel, Switzerland; felix.huth@novartis.com (F.H.); hilmar.schiller@novartis.com (H.S.); 3 Department of Medical Oncology, St Vincent’s University Hospital, D04 T6F4 Dublin, Ireland; 4 Comprehensive Cancer Center Erlangen-EMN, University Hospital Erlangen, 91054 Erlangen, Germany; 5 Interdisciplinary Breast Cancer Center, Helios Klinikum Berlin-Buch, 13125 Berlin, Germany

**Keywords:** drug–drug interaction, early breast cancer, ribociclib, hepatic impairment, renal impairment, CYP3A4

## Abstract

**Background:** Ribociclib, initially approved for HR+/HER2− advanced breast cancer (ABC) at a 600 mg dose, was recently approved for HR+/HER2− early breast cancer (EBC) at a 400 mg dose based on the NATALEE trial. Differences in dose and patient population warrant reassessment of ribociclib drug–drug interactions (DDIs) and the impact of hepatic or renal impairment (HI/RI) in EBC patients to guide co-medication management and subpopulation dose recommendations. **Methods:** Physiologically-based pharmacokinetic (PBPK) modeling based on a healthy volunteer population was conducted to assess ribociclib DDIs with CYP3A4 substrates/modulators in patients with EBC. Subgroup analysis from NATALEE assessed HI/RI impact on ribociclib PK in EBC patients. Existing data from ABC/advanced cancer patients and non-cancer subjects were also integrated to inform dose recommendations for EBC subpopulations. **Results:** PBPK modeling predicted that ritonavir or erythromycin (strong and moderate CYP3A4 inhibitors) would increase ribociclib steady-state area under the concentration–time curve (AUC) by 1.84-fold or show no meaningful impact, respectively. Steady-state ribociclib AUC was estimated to decrease by 83% and 74% with rifampicin and efavirenz, strong and moderate CYP3A4 inducers, respectively. Ribociclib was estimated to increase CYP3A4 substrate midazolam exposure by 280%. Mild HI or mild/moderate RI did not show an apparent impact on ribociclib PK. **Conclusions:** Using relevant data and methodology for EBC patients, this analysis informed the approved ribociclib label of no dose adjustment for EBC patients with concomitant use of a moderate CYP3A inhibitor, any degree of HI, or mild/moderate RI, and a reduced 200 mg dose for patients with concomitant use of a strong CYP3A inhibitor or severe RI.

## 1. Introduction

In 2022, breast cancer was the most commonly diagnosed cancer in women worldwide, accounting for more than 2.3 million new cases and approximately 666,000 deaths [1]. More than 90% of these cases were classified as early breast cancer (EBC) at initial diagnosis, with hormone receptor-positive (HR+), human epidermal growth factor receptor 2-negative (HER2−) disease being the most prevalent subtype [2]. Despite standard-of-care adjuvant endocrine therapy (ET) for 5 years or more with and without chemotherapy for systemic treatment of HR+/HER2− EBC, the risk of recurrence remains a significant issue (5-year overall recurrence: 12.6%, 12.8%, and 33.8% in patients with high-risk node-negative EBC and in those with 1–3 and ≥4 positive lymph nodes, respectively) [3,4]. Thus, new treatments that are more efficacious and have a manageable safety profile are needed to address the unmet need in this patient population [3,5,6].

Ribociclib, an orally bioavailable, highly selective small-molecule inhibitor of cyclin-dependent kinases 4 and 6, directly targets phosphorylation of the retinoblastoma protein to block cell cycle progression [7]. In combination with ET, ribociclib has demonstrated consistent, statistically significant, and clinically meaningful improvement in progression-free survival and overall survival over ET alone in the phase 3 MONALEESA-2, -3, and -7 trials in patients with HR+/HER2− advanced breast cancer (ABC) [8,9,10]. Ribociclib has therefore received global approval for the first- and second-line treatment of pre-/peri-menopausal and post-menopausal women with HR+/HER2− ABC, with a recommended starting dose of 600 mg once daily (QD) for 3 weeks on/1 week off and an option of dose reduction to 400 or 200 mg based on individualized patient safety needs [11,12]. Recently, the results from the pivotal phase 3 NATALEE trial in patients with stage II/III HR+/HER2− EBC with a high risk of recurrence showed a statistically significant and clinically meaningful invasive disease-free survival benefit with ribociclib plus a non-steroidal aromatase inhibitor (NSAI) versus an NSAI alone [13]. This benefit further deepened after all patients discontinued ribociclib treatment [14]. Based on these data, ribociclib was also approved for the adjuvant treatment of high-risk HR+/HER2− stage II/III EBC, with a recommended starting dose of 400 mg QD for 3 weeks on/1 week off and a dose reduction option to 200 mg if needed [11,12].

Ribociclib is primarily eliminated by hepatic metabolism via oxidative pathways mediated by cytochrome P450 3A4 (CYP3A4; 63%) and by flavin-containing monooxygenase 3 (16%), with a small contribution by renal (7%) and intestinal excretion (8%) [15,16,17,18]. Thus, simultaneous use of drugs that modulate CYP3A4 metabolism may impact ribociclib exposure [19]. Additionally, ribociclib is a moderate to strong inhibitor of CYP3A4; thus, avoiding overexposure of drugs that are sensitive CYP3A substrates with a narrow therapeutic index is also important [17].

Findings from prior pharmacokinetic (PK)/pharmacodynamic analyses and clinical pharmacology studies support ribociclib use at the approved 600 mg dose in patients with ABC, with no routine dose adjustments needed; however, individualized dose reductions are recommended for patients with specific medical conditions (moderate to severe hepatic or severe renal impairment or co-medication with a strong CYP3A inhibitor) without compromising safety and efficacy [17,18,19]. The ribociclib starting dose (400 mg) in patients with EBC is lower than the starting dose of 600 mg in patients with ABC. In addition, patients with EBC are generally younger and have fewer comorbidities than patients with ABC [20,21,22]. Our data showed that both dose and population differences account for different PK properties and safety profiles in the two indications [23]. Given that most patients with breast cancer are diagnosed at an early stage, it is critical to reassess the impact of drug–drug interactions (DDIs) and organ impairment on ribociclib exposure in the EBC patient population, which is substantially larger than the ABC patient population. Such assessment will enable accurate characterization of PK profiles in specific subpopulations of patients with EBC and guide individualized prescribing decisions. In this study, DDIs and the effect of organ impairment on ribociclib PK in patients with EBC were assessed to ensure optimal ribociclib dosing recommendations in the prescribing label.

## 2. Results

### 2.1. PBPK Model Selection

**Figure 1:**
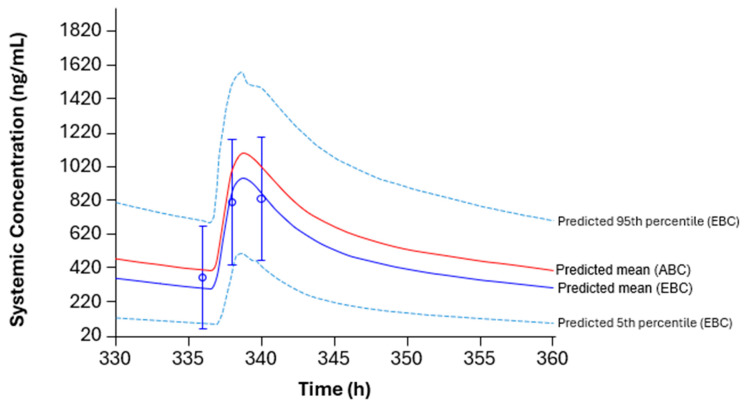
PBPK model-predicted versus observed ribociclib plasma concentrations in the NATALEE trial following daily doses of ribociclib 400 mg on cycle 1, day 15. ABC, advanced breast cancer; EBC, early breast cancer; PBPK, physiologically-based pharmacokinetics. Open circles and error bars represent the observed data (mean and standard deviation). Curves represent PBPK-predicted data.

The physiologically-based pharmacokinetic (PBPK) model was updated based on the previous healthy volunteer (HV) population model developed for ribociclib [17]. As shown in Figure 1, the HV population model matched with the observed data in NATALEE, whereas the cancer patient population model overpredicted ribociclib exposure. Therefore, the HV model was used in the simulation to assess DDIs in patients with EBC [17,24].

### 2.2. Effects of Concomitant Medications on Ribociclib PK

In patients with EBC receiving ribociclib 400 mg (QD for 8 days), the PBPK model-predicted ribociclib steady-state maximum plasma concentration (C_max_) and area under the plasma concentration–time curve (AUC) ratios increased by 1.47- and 1.84-fold, respectively, with co-administration of the strong CYP3A4 inhibitor ritonavir (100 mg twice daily for 8 days) (Table 1). For ribociclib 200 mg (QD for 8 days) co-administered with ritonavir, the PBPK model-predicted ribociclib steady-state C_max_ and AUC ratios were 1.76 and 2.51. In patients receiving ribociclib 400 mg (QD for 8 days) with or without co-administration of the moderate CYP3A4 inhibitor erythromycin (500 mg twice daily for 8 days), no clinically relevant change in ribociclib concentration was predicted (C_max_ ratio of 1.13 and AUC ratio of 1.23) (Table 1).

The strong CYP3A4 inducer rifampicin (600 mg QD for 14 days) decreased ribociclib C_max_ and AUC from time zero to infinity (AUC_inf_) by 66% and 83%, respectively, following multiple doses of ribociclib 400 mg on day 14 [24]. The moderate CYP3A4 inducer efavirenz (600 mg QD for 14 days) was predicted to reduce the exposure of multiple doses of ribociclib 400 mg (on day 14) by 55% (C_max_) and 74% (AUC_inf_) (Table 1) [24].

### 2.3. Effect of Ribociclib on the PK of Concomitant Medications

Ribociclib, dosed at 400 mg QD, is a moderate inhibitor of CYP3A4, and it increased exposure of the CYP3A4 substrate midazolam (2 mg single dose on day 8), by 280% (3.8-fold) after administration for 8 days (Table 2). The model-predicted PK of midazolam co-administered with ribociclib was in line with observed values. Specifically, the observed C_max_ and AUC ratios were 2.05 and 3.75, and the predicted C_max_ and AUC ratios were 2.28 and 4.18.

### 2.4. Effect of Hepatic Function on Ribociclib PK

**Figure 2:**
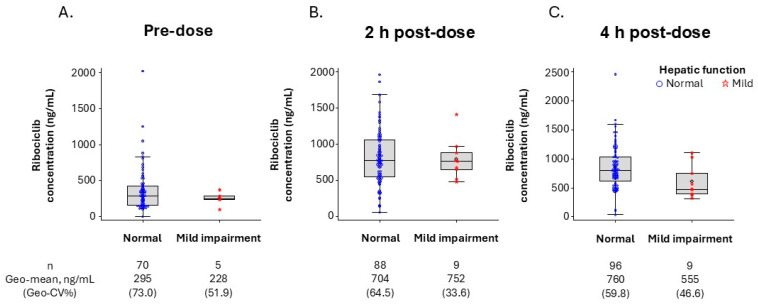
Ribociclib plasma concentrations by hepatic function in the NATALEE trial following daily doses of ribociclib 400 mg on cycle 1, day 15 at pre-dose (**A**), 2 h post-dose (**B**), and 4 h post-dose (**C**). The diamond symbols in the box plots represent the numeric mean.

Among patients with evaluable ribociclib PK data in the NATALEE trial, the geometric mean plasma concentrations of ribociclib at pre-dose (trough concentration [C_trough_]) and 2 and 4 h post-dose in patients with mild hepatic impairment were comparable to those in patients with normal hepatic function. This suggests no apparent impact of mild hepatic impairment on ribociclib PK (Figure 2).

### 2.5. Effect of Renal Function on Ribociclib PK

**Figure 3:**
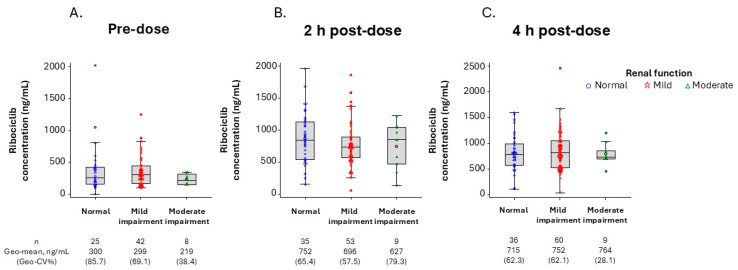
Ribociclib plasma concentrations by renal function in the NATALEE trial following daily doses of ribociclib 400 mg on cycle 1, day 15 at pre-dose (**A**), 2 h post-dose (**B**), and 4 h post-dose (**C**). The diamond symbols in the box plots represent the numeric mean.

Among patients with evaluable ribociclib PK data in NATALEE, the geometric mean plasma concentrations of ribociclib at pre-dose (C_trough_) and 2 and 4 h post-dose in patients with mild and moderate renal impairment were comparable to those in patients with normal renal function, suggesting no apparent impact of mild or moderate renal impairment on ribociclib PK (Figure 3).

In addition, the geometric mean ratios of the population PK (popPK) model-predicted steady-state ribociclib AUC_0–24_ (area under the plasma concentration–time curve over the last 24-h dosing interval), C_max_, and time to maximum plasma concentration (T_max_) in patients with mild renal impairment versus those in patients with normal renal function were 0.994 (90% confidence interval [CI]: 0.895, 1.10), 0.939 (90% CI: 0.853, 1.03), and 1.020 (90% CI: 0.988, 1.05), respectively, suggesting no apparent impact of mild renal impairment on ribociclib PK (Table 3).

## 3. Discussion

Proper understanding of the potential DDIs and impact of organ impairment on ribociclib PK is an important aid for informing labeling recommendations and supporting the safe and effective use of ribociclib in specific subpopulations of patients with EBC, which covers a substantially greater population than patients with ABC. This is particularly important because, even though ribociclib shows a manageable safety profile in both patients with ABC and EBC, adverse events play a key role in the adjuvant setting, where treatment adherence is a major concern [13,25,26,27,28]. In this study, a PBPK model, which was selected based on the NATALEE trial data, was used to simulate different DDI scenarios, and a subgroup analysis based on data from NATALEE was used to assess the impact of organ impairment on ribociclib PK in patients with EBC. These results were integrated with previous data and post-marketing experience in patients with ABC, and they guided dose recommendations in the prescribing label for these subpopulations of patients with EBC.

Two PBPK models were previously developed for ribociclib: the HV population model based on data from HV studies and the cancer patient population model based on data from patients with advanced cancer (Table S2) [17]. The cancer patient population model incorporated PK behavior in patients with advanced cancer, and the cancer population file in Simcyp was revised by reducing the hepatic and intestinal CYP3A4 enzyme abundance to align with the observed data in patients with advanced cancer [17]. To assess DDIs in patients with ABC, a simulation was conducted using the cancer model in a prior publication [17]. However, in patients with EBC, the HV model was used to simulate the steady-state DDI effect on ribociclib PK exposure. This model was used based on the observation that ribociclib PK exposure in patients with EBC in the NATALEE study was comparable to that in HVs but lower than that in patients with ABC at the same 400 mg dose, showing population differences in ribociclib PK [23]. This is plausible, as patients with EBC have less disease burden than patients with ABC, who have more inflammation, which can result in the downregulation of CYP3A4 expression and subsequent lower clearance and higher plasma exposure of ribociclib [23]. Consistent with this observation, PBPK prediction using the HV population model matched with the observed data in the NATALEE trial. Therefore, the HV model was used to predict steady-state DDIs to inform dose recommendations with concomitant medications for patients with EBC. For patients with ABC, the cancer population model was used previously to inform dose recommendations with concomitant medications [17] (Table S2).

At the 400 mg ribociclib dose in patients with EBC, the PBPK model-predicted geometric mean steady-state C_max_ and AUC ratios of ribociclib with versus without co-administration of ritonavir were 1.47 and 1.84, respectively, and the model-predicted geometric mean steady-state C_max_ and AUC of ribociclib with co-administration of ritonavir were 1322 ng/mL and 19,401 ng•h/mL, respectively (Table 1). Based on these data, alternative concomitant medications that have a low potential to inhibit CYP3A4 should be considered in patients with EBC. At the ribociclib dose of 200 mg QD, the geometric mean steady-state C_max_ (661 ng/mL) and AUC (9696 ng•h/mL) values in the presence of ritonavir were below or equivalent to those for the 400 mg dose in the absence of ritonavir (900 ng/mL and 10,523 ng•h/mL, respectively). Therefore, if concomitant use of a strong CYP3A inhibitor is unavoidable, the ribociclib dose should be reduced to 200 mg in EBC patients. For the moderate CYP3A4 inhibitor erythromycin, the geometric mean steady-state C_max_ and AUC ratios for 400 mg ribociclib with versus without co-administration of erythromycin are predicted to be 1.13 and 1.23, respectively, suggesting no apparent effect of moderate CYP3A4 inhibitors on ribociclib PK in EBC patients.

The exposure to ribociclib was markedly reduced when ribociclib was administered with the strong CYP3A4 inducer rifampicin, with decreases of 81% in C_max_ and 89% in AUC_inf_ compared with ribociclib alone, after a single 600 mg oral dose in HVs [17]. Hence, concomitant use of ribociclib with a strong CYP3A4 inducer should be avoided in patients with EBC. Ribociclib dosed at 400 mg QD is a moderate inhibitor of CYP3A4 and increased the exposure to the CYP3A4 substrate midazolam by 280% (3.8-fold) compared with midazolam alone when administered for 8 days in HVs [17]. Therefore, caution is recommended when ribociclib 400 mg is co-administered with CYP3A4 substrates with a narrow therapeutic index in patients with EBC, and dose reduction of sensitive CYP3A4 substrates may be required.

Dose recommendations for ABC patients with hepatic impairment were based on a dedicated phase 1 hepatic impairment clinical pharmacology study in non-cancer subjects and popPK analysis of data from patients with ABC or advanced cancer (Table S3) [18,29]. In the current study, PK data from EBC patients in the NATALEE trial were analyzed to assess the effect of mild hepatic impairment on ribociclib PK. The results showed no apparent increase in ribociclib concentrations in EBC patients, consistent with previous data from non-cancer patients and patients with ABC or advanced cancer [18,29]. Based on these results, no ribociclib dose adjustment is required for EBC patients with mild hepatic impairment (Figure 2) [18].

No PK data from patients with EBC with moderate hepatic impairment were available, and no patients with severe hepatic impairment were enrolled in the NATALEE trial. However, given that the approved starting dose in EBC patients is 400 mg and considering that the benefit–risk profile of the 400 mg starting dose has been established in ABC patients with moderate or severe hepatic impairment through post-marketing experience, no dose adjustment is necessary in EBC patients with moderate or severe hepatic impairment. Unlike no dose reduction recommendation for EBC patients with any degree of hepatic impairment, a dose reduction from 600 to 400 mg is recommended for ABC patients with moderate or severe hepatic impairment (Table S3) [18].

For renal impairment, the dose recommendation for ABC patients with mild or moderate renal impairment was based on data from patients with ABC or advanced cancer, as reported previously [16]. In the current study, the data from EBC patients in the NATALEE trial were analyzed to assess the effect of mild or moderate renal impairment on ribociclib PK. Ribociclib concentrations in EBC patients with mild or moderate renal impairment were comparable to those in patients with normal renal function, suggesting no impact of mild or moderate renal impairment on ribociclib PK. These data are further strengthened by the geometric mean ratios of AUC and C_max_ predicted by the popPK model for EBC patients with mild or moderate renal impairment versus those with normal renal function. Since patients with severe renal impairment were not enrolled in NATALEE, the dose recommendation for severe renal impairment was based on a dedicated phase 1 renal impairment study, where ribociclib C_max_ and AUC in non-cancer subjects with severe renal impairment increased by 130% and 167%, respectively, compared with those with normal renal function. Thus, a lower starting dose of ribociclib (200 mg) is recommended for EBC patients with severe renal impairment [11,12,16]

A limitation of this work is that PK data in moderate/severe hepatic impaired and severe renal impaired patients were not available in the NATALEE trial. This was due to non-enrollment of severe hepatic or renal impaired patients and PK sampling being conducted only from a subset of patients in the NATALEE trial. To overcome this limitation, we integrated data from previous clinical pharmacology studies and ABC patient trials, as well as post-marketing experience in ABC patients. Another limitation of this work is that the population model selected for PBPK simulation was based on only three time points in the NATALEE study. However, the HV population model best describes the observed plasma concentrations, which is also consistent with the observed PK data across populations as described above [23].

Our data show that, with the difference in ribociclib doses and population difference in ribociclib PK, the DDIs and the organ impairment effect can differ across indications; thus, proper PK assessment of DDIs and organ impairment for the relevant indication is critical. To guide the optimal dosing recommendations for the subpopulations of patients with EBC, this analysis was primarily based on the pivotal phase 3 NATALEE trial, which was conducted in the target indication. DDIs and organ impairment PK were previously evaluated and informed the prescribing label for ABC, the initially approved indication for ribociclib [16,17,18]. The current work was based on data from the new indication (EBC), it used different methodology, and it showed that there are differences in ribociclib DDIs and dose recommendations for organ impairment between the ABC and EBC indications, thus adding to the existing literature (Tables S2 and S3). In addition, we leveraged prior data and existing clinical experience to inform and strengthen the labeling decisions for the clinical use of ribociclib in the relevant subpopulations of patients with EBC. Notably, the dose recommendations in EBC patients with DDI scenarios and organ impairment were approved by the health authorities [11,12].

## 4. Methods

### 4.1. NATALEE Study Design

The NATALEE trial was a phase 3, multicenter, randomized, open-label study in men and pre- or post-menopausal women with anatomic stage II or III disease (N = 5101). Patients were randomized 1:1 to ribociclib plus an NSAI or an NSAI alone, with ribociclib administered at 400 mg/day on a 3-weeks-on/1-week-off schedule for 36 months along with an NSAI (either letrozole 2.5 mg/day or anastrozole 1 mg/day) for ≥5 years [30]. Men and pre-menopausal women also received goserelin (3.6 mg once every 28 days). The primary endpoint was invasive disease-free survival, and safety and PK were secondary endpoints. The detailed NATALEE study design has previously been described [30]. Patients with severe hepatic impairment or renal impairment were not enrolled in NATALEE.

For PK analysis, sparse blood samples were collected from a subgroup of patients in the ribociclib arm at pre-dose and at 2 and 4 h post-dose on cycle 1, day 15. Plasma concentrations of ribociclib were measured through a validated liquid chromatography–tandem mass spectrometry assay (lower limit of quantification of 1.00 ng/mL) [31]. Values below this limit were set to zero, and zero concentrations were considered as missing in geometric mean and geometric percent coefficient of variance (%CV) calculations. %CV geometric mean was calculated using the following formula:

$$CV_{geo}\left(\%\right)=\sqrt{\exp\left(\sigma^{2}\right)-1}\times 100\%\;$$

where *σ*^2^ denotes the variance of the log-transformed data.

### 4.2. PBPK Modeling and Simulation to Assess DDIs

Based on the previously developed PBPK models for ribociclib, which included HV and cancer patient population models, the model using the HV population was selected and updated to assess DDIs of ribociclib in patients with EBC [17]. Briefly, this model was developed using available physicochemical data; non-clinical (both in vitro and in vivo) data; previous clinical data from the human absorption, distribution, metabolism, and excretion study in HVs after a single 600 mg oral dose [^14^C] of ribociclib; and DDI studies in HVs with ribociclib as (1) the object drug (co-administered with ritonavir or rifampicin) and (2) the precipitant drug (co-administered with midazolam; precipitant drugs alter the PK of another drug, the object drug) [15,17].

A simulation was conducted on the Simcyp PBPK Simulator version 22 (Certara, Princeton, NJ, USA) using the updated PBPK model to assess CYP3A4-mediated DDIs involving ribociclib as either a precipitant or an object drug. For the DDI of ribociclib as an object drug, the PBPK model was used to estimate the ratios of ribociclib steady-state C_max_ and AUC following multiple doses of ribociclib 400 or 200 mg in the absence and presence of a strong CYP3A4 inhibitor, ritonavir, and a moderate CYP3A4 inhibitor, erythromycin. Ribociclib C_max_ and AUC_inf_ ratios were also predicted in the presence and absence of a strong CYP3A4 inducer, rifampicin, and a moderate CYP3A4 inducer, efavirenz, following multiple doses of ribociclib 400 mg for 14 days. PBPK simulations were generated with 10 iterations, each containing 10 simulated patients (n = 100 per DDI simulated). All individuals were women and ranged in age from 20 to 55 years. For the DDI of ribociclib as a precipitant drug, single-dose midazolam exposure on day 8 in the presence of ribociclib (400 mg once daily for 8 days) was predicted using the updated PBPK model. PBPK predictions were compared with the observed data for midazolam (C_max_, AUC_inf_, and the respective DDI ratios on day 8) obtained from an earlier HV DDI study [17]. The key system- and drug-specific parameters used in the PBPK simulations are described in Table S1.

### 4.3. Hepatic Impairment Assessment

The impact of hepatic function on ribociclib PK was assessed in a subgroup analysis, in which the observed ribociclib PK concentrations on cycle 1, day 15 in the NATALEE study were evaluated by baseline hepatic function. Hepatic function in patients with EBC was determined based on the National Cancer Institute criteria for liver function, with normal hepatic function defined as total bilirubin and aspartate aminotransaminase ≤ upper limit of normal (ULN) and mild hepatic impairment defined as total bilirubin > ULN to 1.5 × ULN or aspartate aminotransaminase > ULN [32].

### 4.4. Renal Impairment Assessment

The impact of renal function on ribociclib PK was assessed in a subgroup analysis of observed ribociclib PK concentrations and in a statistical analysis of model-predicted PK parameters. In the subgroup analysis, the observed PK concentrations of ribociclib on cycle 1, day 15 in the NATALEE study were evaluated through baseline renal function. Renal function was assessed by estimated glomerular filtration rate (eGFR). According to eGFR criteria for kidney disease, normal renal function is defined as an eGFR ≥ 90 mL/min/1.73 m^2^, mild renal impairment is defined as an eGFR of 60–89 mL/min/1.73 m^2^, and moderate renal impairment is defined as an eGFR of 30–59 mL/min/1.73 m^2^ [33].

In the statistical analysis, steady-state PK parameters were predicted through an updated ribociclib popPK model in patients with EBC and compared by their baseline renal function (normal or mild renal impairment) [23]. Briefly, the updated popPK model was used to estimate individual PK parameters (clearance, volume of distribution, and intercompartmental clearance), and results were back-transformed to obtain the adjusted geometric mean, geometric mean ratio, and 90% CIs. For T_max_, the median was determined using ‘adjusted geometric mean’, and difference in median was defined under ‘geometric mean ratio’. A linear model including renal function (categories included normal and mild impairment based on eGFR as a fixed effect) was fitted to the log-transformed PK parameters. The normal cohort was the reference, and point estimates with corresponding 90% CIs were calculated for the mean difference between each test and reference cohort and were anti-logged to obtain the point estimate and 90% CIs.

## 5. Conclusions

This analysis showed that no dose adjustments for ribociclib 400 mg are required in patients with HR+/HER2− EBC when co-administered with a moderate CYP3A4 inhibitor or in EBC patients with mild to moderate renal impairment or any hepatic impairment. A reduced dose of 200 mg is recommended when ribociclib is co-administered with a strong CYP3A4 inhibitor or in EBC patients with severe renal impairment [11]. Integrating data from patients in the new EBC setting, as well as prior data from the previously approved setting, this work effectively guides subpopulation dose recommendations for EBC patients and highlights the importance of using relevant data and methodology to guide prescribing labels for different indications.

## Figures and Tables

**Table 1:** PBPK model-simulated DDI effects of oral administration of ribociclib with different CYP3A4 precipitants in healthy participants.

Precipitant	RIB Dose Regimen	Precipitant Status	Geometric Mean C_max_, ng/mL (%CV)	Geometric Mean AUC, ng•h/mL (%CV)	Geometric Mean C_max_ Ratio (90% CI)	Geometric Mean AUC Ratio (90% CI)
Ritonavir 100 mg BID for 8 days	400 mg QD for 8 days	Without inhibitor	900 (35.5)	10,523 (47.1)	1.47 (1.43, 1.51)	1.84 (1.76, 1.93)
		With inhibitor	1322 (28.9)	19,401 (35.5)		
Ritonavir 100 mg BID for 8 days	200 mg QD for 8 days	Without inhibitor	375 (33.5)	3855 (42.5)	1.76 (1.72, 1.82)	2.51 (2.40, 2.63)
		With inhibitor	661 (28.9)	9696 (35.5)		
Erythromycin 500 mg BID for 8 days	400 mg QD for 8 days	Without inhibitor	900 (35.5)	10,523 (47.1)	1.13 (1.12, 1.14)	1.23 (1.21, 1.24)
		With inhibitor	1021 (33.7)	12,912 (44.0)		
Rifampicin 600 mg QD for 14 days	400 mg QD for 14 days	Without inducer	518 (28.6)	5753 (33.5)	0.342 (0.318, 0.367)	0.171 (0.155, 0.188)
		With inducer	206 (53.6)	1149 (59.2)		
Efavirenz 600 mg QD for 14 days	400 mg QD for 14 days	Without inducer	521 (29.1)	5866 (34.4)	0.449 (0.424, 0.477)	0.260 (0.236, 0.286)
		With inducer	286 (36.2)	1842 (43.1)		

%CV, percent coefficient of variance; AUC, area under the plasma concentration–time curve; BID, twice daily; CI, confidence interval; C_max_, maximum plasma concentration; CYP3A4, cytochrome P450 3A4; DDI, drug–drug interaction; PBPK, physiologically-based pharmacokinetics; QD, once daily; RIB, ribociclib.

**Table 2:** Observed and PBPK model-predicted PK parameters of midazolam following a single oral dose of midazolam with multiple oral doses of ribociclib in healthy participants.

Precipitant Dose Regimen	Object Dose Regimen	Source	Object Drug Geometric Mean C_max_ Ratio (90% CI)	Object Drug Geometric Mean AUC Ratio (90% CI)
RIB 400 mg QD for 8 days	Midazolam 2 mg SD on day 8	Observed	2.05 (1.88, 2.23)	3.75 (3.41, 4.11)
		Predicted	2.28 (2.18, 2.38)	4.18 (3.84, 4.55)

AUC, area under the plasma concentration–time curve; CI, confidence interval; C_max_, maximum plasma concentration; PBPK, physiologically-based pharmacokinetics; PK, pharmacokinetics; QD, once daily; RIB, ribociclib; SD, single dose.

**Table 3:** Statistical analysis of steady-state ribociclib PK parameters by renal function in the NATALEE trial following 400 mg dose of ribociclib.

Model-Predicted PK Parameter	Cohort	n	Adjusted Geometric Mean	Cohort Comparison			
				Comparison	Geometric Mean Ratio	90% Lower CI	90% Upper CI
C_max_, ng/mL	Normal	45	1050	Mild impairment/normal	0.939	0.853	1.03
	Mild impairment	69	991				
AUC_0–24_, ng•h/mL	Normal	45	10,500	Mild impairment/normal	0.994	0.895	1.10
	Mild impairment	69	10,400				
T_max_, h	Normal	45	3.63	Mild impairment/normal	1.020	0.988	1.05
	Mild impairment	69	3.70				

AUC_0–24_, area under the plasma concentration–time curve over the last 24-h dosing interval; CI, confidence interval; C_max_, maximum plasma concentration; PK, pharmacokinetics; T_max_, time to maximum plasma concentration. For T_max_, the median is presented under ‘Adjusted geometric mean’ and the difference in the median is presented under ‘Geometric mean ratio’.

## Data Availability

The original contributions presented in this study are included in the article/Supplementary Material. Further inquiries can be directed to the corresponding author.

## Supplementary Materials

The following supporting information can be downloaded at: https://www.mdpi.com/article/10.3390/ph19030461/s1. Table S1. Parameter values used for ribociclib simulations; Table S2. Comparison of ribociclib PBPK modeling & simulation for DDI assessment in ABC vs. EBC patients; Table S3. Comparison of hepatic impairment and renal impairment assessment in ABC vs. EBC patients. References [11,12,16,17,18,29] are cited in the Supplementary Materials.
