# Supplementary material for: Physiologically-Based Pharmacokinetics of Ribociclib Drug–Drug Interactions and Organ Impairment Pharmacokinetics in Early Breast Cancer
Source: Pharmaceuticals (Basel). 2026 Mar 11;19(3):461. doi: 10.3390/ph19030461 (PMC13029677; doi:10.3390/ph19030461)
Supplement: Supplementary file 1 [file pharmaceuticals-19-00461-s001.zip › pharmaceuticals-4081230-supplementary.pdf]

Table S1. Parameter values used for ribociclib simulations

Parameter	Description	Unit	Value	Comment
Physiological and binding properties				
MW	Molecular weight	g/mol	434.54	
Log P	Octanol-water partition	-	1.954	
Compound type	-	-	Diprotic base	
pKa1/pKa2	-	-	8.52 / 5.63	Samant et al 2020
B/P ratio	Blood to plasma drug concentration ratio	-	1.01	
fu	Fraction unbound in plasma	-	0.30	
Main plasma binding protein	-	-	Human serum albumin	
Absorption				
Absorption model	First order absorption model (ADAM[1])	-	-	
fa	Fraction available from dosage form	-	1.00 (0.942[1])	assumed
CV fa	Coefficient of variation fa	%	30	[2]
ka	Absorption rate constant	1/h	0.700 (1.052[1])	optimized
CV ka	Coefficient of variation ka	%	30	[2]
Lag time	-	h	1.10 (0.00[1])	optimized
fu(gut)	Unbound fraction in enterocytes	-	1	
Q(gut)	Nominal flow in gut model	L/h	10.9	
CV Q(gut)	Coefficient of variation Q(gut)	%	30	Samant et al 2020
Papp Caco-2	Caco-2 permeability (apical to basal)	10^{-6} cm/s	1.78	
Papp propranolol	Reference compound permeability	10^{-6} cm/s	3.48	
Distribution				
PBPK model	-	-	Full PBPK – Method 2	
Tissue model	-	-	Perfusion limited	
Vss	Volume of distribution at steady-state	L/kg	13.3	Samant et al 2020

CV Vss	Coefficient of variation Vss	%	30	[2]
Kp scalar	Correction factor for Vss	-	3.26	optimized
Enzyme phenotyping (human liver microsomes; HLM)				
Vmax (CYP3A4)	*In vitro* maximum enzyme velocity	pmol/min/mg	318	
Km (CYP3A4)	*In vitro* Michaelis-Menten constant	μM	6.67	Samant et al 2020
fu(inc) (CYP3A4)	Fraction unbound *in vitro*	-	1.0	
Other distribution and elimination properties				
In vivo CL	Total clearance	L/h	40.2	Samant et al 2020
CLr	Renal clearance	L/h	1.22	Samant et al 2020
In vitro CL				
Uptake hep	Total hepatic uptake clearance / passive diffusion in hepatocytes	-	1.0	
HLM CLint	Additional undefined HLM CLint	μL/min/mg	16.8[3]	
CV HLM CLint	Coefficient of variation HLM CLint	%	30	Samant et al 2020
fu(inc) in HLM	Fraction unbound in HLM incubates	-	1.0	
CLint(hep)	Overall biliary clearance	μL/min/10^6 cells	0	
CV CLint(hep)	Coefficient of variation CLint(hep)	%	30	
Interaction				
CYP inhibition (competitive)				
Ki (CYP1A2)	Inhibition constant	μM	13.1	Samant et al 2020
fu(inc) (CYP1A2)		-	1.0	
Ki (CYP3A)		μM	35	Samant et al 2020
fu(mic) (CYP3A)		-	0.86	
CYP inhibition (time-dependent)				
KI (CYP3A)	Inhibition constant	μM	8.60	Samant et al 2020
kinact (CYP3A)	Inactivation rate of enzyme	1/h	1.0	

fu(inc) (CYP3A)	-	1.0

[1] Parameters of the previous PBPK model of Samant et al, Clin Pharm Ther 2020 108 (3): 575-585.

[2] For CV(%) values of input parameters, the Simcyp default value (30%, Version 22) was used, unless noted.

[3] Hepatic intrinsic clearance due to FMO3-mediated metabolism is represented.

Table S2. Comparison of ribociclib PBPK modeling & simulation for DDI assessment in ABC vs. EBC patients

**Parameter**	**PBPK model in ABC (Samant et al, 2020)**	**PBPK model in EBC (current study)**
Ribociclib doses investigated	400, 600 mg SD with HV<br>200, 400, 600 mg QD with ABC	200, 400 mg QD with EBC (HV)
Populations investigated	HV and ABC	EBC (HV)
PBPK models	Ribociclib compound model, HV and cancer population models in Simcyp V18	Ribociclib compound model, EBC (HV) population models in Simcyp V22[1]
DDI simulation	To inform dose recommendation:<br>RIB 200 mg QD + RTV 100 mg BID with ABC<br>RIB 200 mg QD + KET 200 mg BID with ABC<br>RIB 400 mg QD + RTV 100 mg BID with ABC<br>RIB 400 mg QD + ERY 500 mg BID with ABC<br>RIB 600 mg QD + RTV 100 mg BID with ABC<br>RIB 600 mg QD + ERY 500 mg BID with ABC<br>RIB 600 mg QD + FLU 50 mg QD with ABC<br>RIB 600 mg QD + RIF 600 mg QD with ABC	To inform dose recommendation:<br>RIB 200 mg QD + RTV 100 mg BID with EBC<br>RIB 400 mg QD + RTV 100 mg BID with EBC<br>RIB 400 mg QD + ERY 500 mg BID with EBC<br>RIB 400 mg QD + RIF 600 mg QD with EBC<br>RIB 400 mg QD + EFA 600 mg QD with EBC

	RIB 600 mg QD + EFA 600 mg QD with ABC<br>To validate the model:<br>RIB 400 mg SD + RTV 100 mg BID with HV<br>RIB 600 mg SD + RTV 100 mg BID with HV<br>RIB 600 mg SD + RIF 600 mg QD with HV<br>RIB 600 mg SD + EFA 600 mg QD with HV	

ABC: Advanced breast cancer, BID: Twice daily, EBC: Early breast cancer, HV: Healthy volunteer, KET: ketoconazole, EFA: Efavirenz, ERY: Erythromycin, FLU: Fluvoxamine, Ketoconazole, QD: Once daily, RIF: Rifampicin, RTV: Ritonavir
[1] Model parameters and differences from the prior published model by Samanth et al, Clin Pharm Ther 2020 are described in Table S1

Table S3. Comparison of hepatic impairment and renal impairment assessment in ABC vs. EBC patients

		ABC (starting dose :600 mg)	EBC (starting dose: 400 mg)
Hepatic impairment	Data used for dose recommendation	• Hepatic impairment study in non-cancer subjects[1]<br>• PopPK analysis of data from patients with ABC and advanced cancer[2]	• Subgroup analysis of data from EBC patients<br>• Dose recommendation in ABC
	Dose recommendation	• No dose adjustment is required in mild hepatic impairment; a reduced starting dose of 400 mg once daily is recommended in moderate or severe hepatic impairment[3,4]	• No dose adjustment is required in patients with hepatic impairment[3,4]

Renal impairment	Data used for dose recommendation	• Subgroup analysis of data from patients with ABC and advanced cancer[5]<br>• Renal impairment study in non-cancer subjects (for severe renal impairment)[5]	• Subgroup analysis of data from EBC patients<br>• Renal impairment study in non-cancer subjects (for severe renal impairment)[5]
	Dose recommendation	• No dose adjustment is required in mild or moderate renal impairment; a reduced starting dose of 200 mg once daily is recommended in severe renal impairment[3,4]	• No dose adjustment is required in mild or moderate renal impairment; a reduced starting dose of 200 mg once daily is recommended in severe renal impairment[3,4]

ABC, advanced breast cancer; EBC, early breast cancer. 1. Samant TS, et al. *The Journal of Clinical Pharmacology*. 2021; 61(8):1001-1009. 2. Lu Y, et al. *J Clin Pharmacol*. 2021;61(8):1054-1068. 3. Kisqali prescribing information. https://www.novartis.com/us-en/sites/novartis_us/files/kisqali.pdf. 4. Kisqali. prescribing information Accessed 5/20/25, 2025. https://www.ema.europa.eu/en/medicines/human/EPAR/kisqali.5. Ji Y, et al.. *Clin Pharmacokinet*. 2023;62(3):493-504.
